# In Search of Emerging Same-Sex Sexuality: Romantic Attractions at Age 13 Years

**DOI:** 10.1007/s10508-016-0726-2

**Published:** 2016-04-18

**Authors:** Gu Li, Melissa Hines

**Affiliations:** Department of Psychology, University of Cambridge, Free School Lane, Cambridge, CB2 3RQ UK

**Keywords:** Sexual orientation, Sexuality, Development, Sex-typed behavior, Early adolescence, ALSPAC

## Abstract

Sex-typed behavior in childhood is significantly related to sexual orientation in adulthood. In addition, same-sex attractions in early adolescence are more non-exclusive than in adulthood and can differ from later same-sex orientations. However, little research has focused on romantic attractions as they emerge during early adolescence. Drawing a sample from the Avon Longitudinal Study of Parents and Children (197 girls, 204 boys), the current study examined whether same-sex romantic attractions at age 13 years were exclusive, and whether they were predicted by sex-typed behavior at age 3.5 years. No young adolescents in this sample reported exclusive same-sex attractions, and increased same-sex attractions were not significantly related to reduced other-sex sexualities. Childhood sex-typed behavior did not significantly predict early same-sex attractions, suggesting that early same-sex attractions differ from later same-sex orientations. The current study highlights the importance of studying the development of sexuality beginning prior to adulthood.

**Electronic supplementary material:**

The online version of this article (doi:10.1007/s10508-016-0726-2) contains supplementary material, which is available to authorized users.

## Introduction

The development of sexuality is one of the most salient aspects of adolescence. From age 10 years, children begin to experience romantic or sexual attractions (D’Augelli, Grossman, Starks, & Sinclair, 2010; McClintock & Herdt, 1996; Savin-Williams & Diamond, 2000). Whereas the majority of children and young adolescents are exclusively attracted toward the other sex, some are attracted toward the same sex. These early attractions are presumably milder and more transient than attractions at later developmental stages, but they are likely to be developmentally significant (Diamond, Bonner, & Dickenson, 2015). Describing these early same- and other-sex attractions and their relations to sexual activities and cognitions could help elucidate sexual development more generally.

Limited research has examined romantic attractions as they emerge during early adolescence. Previous studies documented the timing and sequence of the milestone events (e.g., first same-sex attractions, first other-sex sexual activity, etc.) (studies reviewed in Fox, 1995; Calzo, Antonucci, Mays, & Cochran, 2011; D’Augelli et al., 2010; Herdt & Boxer, 1996; Savin-Williams & Diamond, 2000). However, these studies are based on sexual minority adults’ self-recall of early experiences and thus could have excluded many same-sex attracted young adolescents who would eventually identify as heterosexual.

Contemporaneous reports of young adolescents offer additional insight into early same-sex sexuality (e.g., Austin et al., 2009; Remafedi, Resnick, Blum, & Harris, 1992). Two themes emerge from these studies: (1) early same-sex attractions are predominantly non-exclusive, and (2) early same-sex attractions can differ from later same-sex sexual orientations.

### Non-Exclusivity of Emerging Same-Sex Sexuality

The term non-exclusive sexuality has been used to refer to one or more of four concepts: (1) at a population level, the percentage of people reporting non-exclusive attractions, sexual behaviors, or sexual identities (e.g., mostly heterosexual, bisexual, mostly lesbian/gay, questioning, or unlabeled); (2) at an individual level, the relation between same- and other-sex sexuality in the same domain, with a non-negative correlation indicating non-exclusive sexuality (e.g., an increased intensity of same-sex attractions is not associated with a decreased intensity of other-sex attractions); (3) again at an individual level, agreement across dimensions of sexuality (e.g., attractions with behaviors); and (4) also at the individual level, change within a given domain of sexuality over time.

Most studies of non-exclusive sexuality are based on adults. Women are consistently found to have more non-exclusive sexuality than do men for the first three concepts. National surveys in Western countries have found that a larger proportion of women than men self-report non-exclusive sexual identities and a smaller proportion self-report exclusive ones (reviewed in Savin-Williams & Vrangalova, 2013). In addition, women’s same- and other-sex sexual arousal and attractions are not negatively correlated, whereas men’s are (Chivers, Rieger, Latty, & Bailey, 2004; Lippa, 2006, 2007). Further, women’s sexual responses are less consistent across measures of sexuality than are men’s (Chivers, Seto, Lalumière, Laan, & Grimbos, 2010; Rieger et al., 2015; Savin-Williams, 2014).

A few studies have examined non-exclusive sexuality in early adolescence. Their results suggest that more sexual minority adolescents were non-exclusively attracted to both sexes than were exclusively attracted to the same sex (Austin et al., 2009; Busseri, Willoughby, Chalmers, & Bogaert, 2006; Saewyc, Poon, Homma, & Skay, 2008; Saewyc, Skay, Bearinger, Blum, & Resnick, 1998; but see Remafedi et al., 1992, for an exception). In addition, there may be a sex difference in the prevalence of non-exclusive sexuality: among adolescents aged 12–14 years, girls were found to be 1.7 times more likely than boys to report non-exclusive attractions (Austin et al., 2009). Also, the percentage of non-exclusive attractions among sexual minority youth declined with age, with a seemingly larger decrease in boys (from 90 % in the 12–14 age group to 72 % in the 21–23 age group) than in girls (from 99 to 92 % in corresponding groups) (Austin et al., 2009). This suggests that non-exclusive attractions may be more prevalent in early adolescence than in later years, regardless of sex, but that boys more than girls become increasingly exclusively attracted over time.

However, no prior studies have examined other aspects of non-exclusive sexuality in early adolescence, including the relations between the intensities of same- and other-sex attractions at an individual level. Also seldom studied is how emerging same-sex attractions relate to other sexuality components, such as other-sex sexual activities and heterosexual expectations—expectations of leading a heteronormative life, including getting married and having babies with an other-sex partner (Carver, Egan, & Perry, 2004).

### Same-Sex Attractions or Same-Sex Sexual Orientations? Relation With Childhood Sex-Typed Behavior

Related to the preponderant non-exclusivity of emerging same-sex attractions, a question commonly asked is whether same-sex attractions in early adolescence represent one’s same-sex sexual orientation or a transient phase. The question relates to the underlying assumption that a person has a core sexual orientation that, although not always fully expressed, stays relatively stable over time (Bailey, 2009; Diamond, 2008; Farr, Diamond, & Boker, 2014).

One approach to this question is to examine how emerging same-sex attractions are associated with constructs that closely relate to sexual orientation. One construct that relates to sexual orientation is sex-typed behavior in childhood. It is well established that gender nonconformity in childhood—reduced interests in same-gender toys and activities and/or heightened interests in cross-gender toys and activities—is linked to same-sex sexuality in adulthood. This relation has been observed in retrospective studies comparing lesbian/gay and heterosexual adults (Bailey & Zucker, 1995; Rieger, Linsenmeier, Gygax, & Bailey, 2008; see also Zucker, 2008) and in prospective studies of clinically referred extremely gender nonconforming children (Drummond, Bradley, Peterson-Badali, & Zucker, 2008; Green, 1987; Singh, 2012; Wallien & Cohen-Kettenis, 2008; see also Zucker & Bradley, 1995).

In a prospective study that followed children into adulthood in a general population sample (Steensma, van der Ende, Verhulst, & Cohen-Kettenis, 2013), children who had cross-gender behavior or wishes (about 6 % of the sample) were 8.4–15.8 times more likely to report a same-sex sexual orientation in adulthood than children who did not have such behavior or wishes. Further, the difference in sex-typed behavior between pre-lesbian/gay and pre-heterosexual children may appear before age 5 years (Rieger et al., 2008). Therefore, if children from the general population who are gender nonconforming in early childhood are followed into adulthood, it is likely that more of these individuals than others will have a same-sex sexual orientation; if sexual orientation is expressed in attractions in early adolescence, childhood gender nonconforming behavior should also predict same-sex attractions among adolescents and, among sexual minority youth, increased intensity of same-sex attractions.

### The Current Study

The current study investigated the characteristics of same-sex romantic attractions in 13-year-old girls and boys. It assessed children’s sex-typed behavior at 3.5 years in a general population and then drew a sample of gender conforming, gender nonconforming, and control children. When the selected children reached 13 years, the study measured their same- and other-sex romantic attractions, other-sex sexual activities, and heterosexual expectations. Based on prior research, the following hypotheses were evaluated:

1. At the population level, non-exclusive same-sex romantic attractions are more prevalent than exclusive same-sex romantic attractions. Further, a larger percentage of girls than boys will report non-exclusive romantic attractions.
2. At an individual level, the intensities of same- and other-sex attractions will be either positively correlated or not significantly correlated among non-exclusively attracted girls. Among non-exclusively attracted boys, however, the correlation will be negative. Boys who have any same-sex attractions will report significantly fewer other-sex sexual activities or heterosexual expectations than boys who report exclusive other-sex attractions, whereas girls who have any same-sex attractions will not report significantly fewer other-sex sexual activities or heterosexual expectations than girls who report exclusive other-sex attractions. Among same-sex attracted youth, the intensity of same-sex attractions will relate negatively to other-sex sexual activities and heterosexual expectations in boys, but not in girls.
3. Adolescents who were gender nonconforming in early childhood will be more likely than others to report same-sex romantic attractions. Further, among same-sex attracted youth, childhood gender nonconforming behavior will positively predict same-sex romantic attractions.

## Method

### Participants

The sample was part of the Avon Longitudinal Study of Parents and Children (ALSPAC), which attempted to enroll all pregnant women between 1991 and 1992 within Avon, in southwest England. Initial recruitment included 14,541 pregnant women (71.8 % of the eligible population), who delivered 14,062 live births. For more details, see Boyd et al. (2013) and Golding, Pembrey, Jones, and the ALSPAC Study Team (2001).

At age 3.5 years, children’s sex-typed behavior was assessed using the Pre-School Activities Inventory (PSAI), a psychometrically standardized measure designed specifically to distinguish differences in gender (non)conformity within each sex among children ages 2–7 years (Golombok & Rust, 1993a, 1993b). Based on PSAI scores, six groups of children were identified in the ALSPAC sample: gender conforming boys and girls, gender nonconforming boys and girls, and control boys and girls randomly selected from the remaining child cohort. See Table 1 for descriptive statistics for the six groups of children.

**Table 1:** Descriptive statistics of Pre-School Activities Inventory scores for children classified as gender conforming, control, or gender nonconforming at age 3.5 years and in the same groups of children available for follow-up at age 13 years

	Boys					Girls				
	*n*	*M*	*SD*	95 % CI	Range	*n*	*M*	*SD*	95 % CI	Range
At 3.5-year sampling										
Gender conforming children	122	80.68	4.07	79.95, 81.41	75.75–95.55	109	17.10	4.16	16.31, 17.89	4.25–21.85
Control children	99	62.02	6.13	60.79, 63.24	49.35–74.65	108	34.84	7.51	33.40, 36.27	22.95–49.35
Gender nonconforming children	110	43.96	4.61	43.09, 44.83	20.75–48.25	111	56.91	4.58	56.05, 57.77	51.55–71.35
At 13-year follow-up										
Gender conforming children	66	80.70	4.60	79.57, 81.83	75.75–95.55	69	17.12	4.14	16.12, 18.11	5.35–21.85
Control children	56	61.19	5.90	59.61, 62.78	49.35–74.65	67	35.31	7.72	33.43, 37.20	22.95–49.35
Gender nonconforming children	82	43.98	4.43	43.00, 44.96	20.75–48.25	61	56.80	4.76	55.58, 58.02	51.55–70.25

*Note* Higher PSAI scores represent more masculine behavior and/or less feminine behavior. The standardized norm is *M* = 60, *SD* = 10 for boys and *M* = 40, *SD* = 10 for girls (Golombok & Rust, 1993a, 1993b). The absolute range of the PSAI scores is −4.55 to 101.05

Between 2004 and 2005, when the six groups of children reached 13 years of age, they were invited to join a follow-up study about their psychological development. A total of 401 children participated in the follow-up study (Table 1), although the sample size varied per analysis due to deletions of missing data (less than 10 %; see Results). Little’s (1988) MCAR test indicated that the missingness was completely at random, χ^2^(59, *N* = 401) = 68.19, *p* = .19, suggesting that the missing data due to participant non-responses should not bias the results. The participation rate was 61 % of the 3.5-year sample, slightly higher than the rate of 54 % of adolescents from the entire ALSPAC cohort who took part in other follow-up assessments in the same year. There was no differential withdrawal among groups of girls, χ^2^(2, *n* = 328) = 1.06, or between gender conforming and control boy groups, χ^2^(1, *n* = 221) < 1. However, gender nonconforming boys were more likely to participate at age 13 years than gender conforming boys, χ^2^(1, *n* = 232) = 9.60, *p* = .002, or control boys, χ^2^(1, *n* = 209) = 6.73, *p* = .009. The follow-up sample was representative of Avon, southwest England and diverse in socioeconomic background: About 50 % of the parents had an occupation of professional/managerial/technical and 50 % skilled/partly skilled/unskilled. The majority (96 %) of the children were Caucasian.

At age 13 years, participants were asked about their same- and other-sex romantic attractions, their other-sex sexual activities, and their heterosexual expectations. Pubertal development was also assessed, for control purposes. Two strategies were used to enhance the quality of self-reporting of sensitive information: First, the measures related to sexuality were embedded within a larger study protocol that also included numerous measures of gender-related cognitive and personality characteristics and of family and peer relationships, so that the sexuality measures were not the main focus of the study; second, adolescents completed the questionnaires on a computer by themselves and in privacy (Turner et al., 1998).

### Measures

#### Childhood Sex-Typed Behavior

Caregivers rated their child’s sex-typed behavior using the PSAI (Golombok & Rust, 1993a, 1993b). The PSAI includes 12 masculine and 12 feminine items assessing children’s toy preferences (e.g., tea set [feminine]), activity preferences (e.g., playing house [feminine]), and characteristics (e.g., enjoys rough-and-tumble play [masculine]). Scoring used the standardized scoring protocol. Each item was scored using a 5-point scale ranging from “never” to “very often.” Feminine items were reverse-scored and combined with masculine items to form a composite score of childhood sex-typed behavior. Thus, higher total scores indicate more masculine behavior and less feminine behavior for both boys and girls. The PSAI demonstrated good internal consistency in the current sample, α = .94. Based on PSAI scores, boys and girls were classified into gender conforming, gender nonconforming, and control groups (Table 1).^1^ PSAI scores of gender nonconforming boys were 1.2 *SD*s to 3.9 *SD*s below the boys’ standardized norm of *M* = 60 and *SD* = 10; scores of gender nonconforming girls were 1.2 *SD*s to 3.0 *SD*s above the girls’ standardized norm of *M* = 40 and *SD* = 10 (Golombok & Rust, 1993a, 1993b).

#### Adolescent Romantic Attractions

Adolescents’ romantic attractions were measured using a 14-item questionnaire adapted from the Erotic Response and Orientation Scale (Storms, 1980). Participants described their romantic experiences and feelings over the past 6 months, toward the same sex and the other sex, on a 5-point Likert scale (0 = not at all, 4 = almost every day). The experiences and feelings asked about included being attracted to someone, touching someone, and daydreaming about someone (Appendix). Two items from the original Storms’ scale inquiring about masturbation were not used due to concerns about the possibility of alienating families. Composite mean scores were calculated separately for other-sex romantic attractions and for same-sex romantic attractions, with higher scores representing more frequent attractions. The two sub-scales showed good reliability; αs for other- and same-sex attractions in the current sample were .91 and .82, respectively. In addition, confirmatory factor analyses demonstrated that items loaded separately on other- and same-sex sub-scales; measurement equivalence was also established between girls and boys (Appendix). Because the distribution of scores for same-sex romantic attractions was not normal, and statistical transformations could not fully adjust the non-normal distribution, bootstrapped 95 % confidence intervals (CIs; bias-corrected and accelerated) were calculated, after 1000 resampling iterations, for parametric analyses involving the intensity of same-sex romantic attractions.

In addition, non-attracted adolescents were defined as those who scored 0 on both subscales, exclusively other-sex attracted youth were defined as those who scored 0 on same-sex subscale but higher than 0 on other-sex subscale, and same-sex attracted youth were defined as those who scored higher than 0 on the same-sex subscale. Among those who were same-sex attracted, a distinction was made between adolescents who reported non-exclusive attractions (scored higher than 0 on the other-sex subscale) from those who reported exclusive same-sex attractions (scored 0 on the other-sex subscale).

#### Adolescent Other-Sex Sexual Activities

Other-sex sexual activities were assessed using the Adolescent Sexual Activities Index (Hansen, Paskett, & Carter, 1999). Twelve sexual activities were categorized into five stages based on the intensity of the activity: “Hugging,” “holding hands,” “spending time alone,” “kissing on the mouth,” and “being kissed on the mouth” were in the first stage, “cuddling,” “laying down together,” “being touched under clothes,” and “touching under clothes” in the second, “being undressed with private parts showing” in the third, “touching or foundling a boy’s/girl’s private parts” in the fourth, and “having sexual intercourse” in the fifth stage. Adolescents reported whether or not they had each of the experiences; if they answered “no” to all the items in one stage, they did not progress to the next stage. Items were fitted using the Rasch model, a one-parameter logistic model derived from item response theory (IRT) (Embretson & Reise, 2000); higher scores reflect a more advanced stage of other-sex sexual activities. See Appendix for more information on IRT scoring.

#### Adolescent Heterosexual Expectations

Adolescents reported their expectations for future romantic involvement with other-sex persons on the 5-item Heterosexual Identity sub-scale of the Multidimensional Gender Identity Scale (Carver et al., 2004). Items assessing heterosexual expectations asked about expectations of falling in love and getting married with a person of the other sex, having a family when grown up, and becoming a parent one day. Item scores were averaged to form a composite heterosexual expectation score for each participant; higher scores represent more heterosexual expectations. The scale demonstrated good internal consistency in this sample, α = .85.

#### Pubertal Development

Adolescents reported their pubertal development status using line drawings developed by Morris and Udry (1980). The instrument consisted of two sections: pubic hair development and physical development (female breasts, male genitalia, and testicular size), and each section contained five drawings representing Tanner’s (1962) five stages of pubertal development. Participants were asked to choose the drawing that was closest to their current stage, with higher scores indicating more advanced stages. Because pubic hair development best represents adrenarche, and physical development best represents gonadarche (Dorn & Biro, 2011), the two scores were not combined but instead were kept separate in analyses. Pubertal development was controlled in subsequent multivariate analyses, because prior research suggests that adrenarche relates to the genesis of romantic or sexual attractions (McClintock & Herdt, 1996). However, this control was not used when the sample was restricted to same-sex attracted adolescents because of reduced statistical power (Tabachnick & Fidell, 2001).

## Results

### Romantic Attractions: Preliminary Analyses

About 6 % (*n* = 22) of participants reported neither same- nor other-sex romantic attractions during the past 6 months. A 2 (participant sex: male or female) × 2 (no attractions: no or yes) chi square test indicated that the percentages of non-attracted youth did not differ by participant sex, χ^2^(1, *n* = 392) = 1.80. This finding indicated that most adolescents experienced romantic attractions at age 13 years. Another 81 % (*n* = 318) of participants experienced exclusively other-sex romantic attractions; there was no significant sex difference in the percentages of exclusively other-sex attracted youth, χ^2^(1, *n* = 392) = 2.55, *p* = .11. Another 13 % (*n* = 52) of adolescents reported same-sex romantic attractions; there was no significant sex difference in the percentages of same-sex attracted youth, χ^2^(1, *n* = 392) < 1.

Notably, same-sex romantic attractions were always accompanied by other-sex romantic attractions. That is, despite our over-sampling of gender nonconforming children, no 13-year-old girls or boys reported exclusively same-sex romantic attractions. Further, the non-exclusively attracted adolescents reported significantly higher mean levels of romantic attractions to the other sex than to the same sex, paired-sample *t*(51) = 12.66, *M* of difference = 1.53, *p* < .001, bootstrapped 95 % CI of difference = [1.30, 1.78]. Moreover, each of these 52 adolescents reported a higher level of other-sex romantic attractions than same-sex romantic attractions. Similarly, among all the adolescents, the maximum level of other-sex romantic attractions was higher than that of same-sex romantic attractions (Table 2). Finally, among the non-exclusively attracted adolescents, same- and other-sex attractions were positively correlated in boys, *r*(23) = .47, *p* = .02, bootstrapped 95 % CI of *r* = [.01, .88], and in girls, *r*(29) = .37, *p* = .05, bootstrapped 95 % CI of *r* = [−.02, .66]. The overlapping confidence intervals indicated no sex difference in the correlations between same- and other-sex attractions. These results regarding the intensity of same- and other-sex attractions did not significantly change after controlling for sampling group. Therefore, of necessity, same-sex attracted adolescents in the subsequent analyses included non-exclusively attracted adolescents only, because no adolescents were exclusively attracted to the same sex.

**Table 2:** Descriptive statistics collected at age 13 years (*N* = 401)

	Boys					Girls				
	*n*	*M*	*SD*	95 % CI	Range	*n*	*M*	*SD*	95 % CI	Range
Same-sex romantic attractions	197	0.04	0.23	0.02, 0.08^a^	0 to 2.71	195	0.06	0.20	0.03, 0.09^a^	0 to 1.14
Other-sex romantic attractions	197	1.71	0.99	1.57, 1.85	0 to 4	195	1.40	0.91	1.27, 1.53	0 to 4
Heterosexual expectations	194	3.12	0.65	3.03, 3.22	1 to 4	187	3.11	0.66	3.02, 3.21	1 to 4
Other-sex sexual activities	204	0.07	0.94	−0.06, 0.20	−1.08 to 2.62^b^	197	0.01	0.90	0.12, 0.13	−1.08 to 2.62^b^
Pubic hair development	186	3.35	0.97	3.21, 3.50	1 to 5	186	3.83	0.84	3.71, 3.95	1 to 5
Physical development	185	3.61	0.96	3.47, 3.74	2 to 5	188	3.49	0.87	3.36, 3.62	1 to 5

*Note* A higher value of a variable represents a larger extent or a higher level of that variable. Overlapping CIs between the sexes indicate significant sex differences at α = .05, two-tailed

^a^Bootstrapped 95 % CI is reported

^b^IRT scores as generally used are standardized so that they can vary from −∞ to +∞, although in practical this range changes depending on item difficulty in a particular scale (how many participants scored 0 versus 1 on an item) (Embretson & Reise, 2000). These scores can be used to infer the probability of a participant engaging in a given sexual activity (cf. Fig. A, upper panel)

### Same-Sex Attractions and Other-Sex Sexual Activities and Heterosexual Expectations

The next set of analyses examined whether increased same-sex romantic attractions were significantly related to decreased other-sex sexual activities or heterosexual expectations in boys and/or girls. This hypothesis was tested in two ways: First, in ANOVAs, sampling group (gender nonconforming, gender conforming, or control) and romantic attraction group (same-sex attracted or exclusively other-sex attracted) were entered to predict other-sex sexual activities and heterosexual expectations. Second, restricted to same-sex attracted youth (in this study, non-exclusively attracted adolescents), same-sex and other-sex attractions were used to predict the two other sexuality-related outcomes in multivariate regression analyses. The effect of sampling groups was not controlled in these regression analyses due to the low statistical power provided by the small numbers of non-exclusively attracted boys and girls.

In boys, the main effect of sampling group (gender nonconforming, gender conforming, or control) on other-sex sexual activities, *F*(2, 191) < 1, *p* = .78, the main effect of romantic attraction group (same-sex attracted or exclusively other-sex attracted), *F*(1, 191) = 1.23, *p* = .27, and their interaction, *F*(2, 191) = 0.13, *p* = .88, were all not significant. Similar non-significant findings were observed in girls for the main effect of sampling group on other-sex sexual activities, *F*(2, 189) = 2.15, *p* = .12, the main effect of romantic attraction groups, *F*(1, 189) = 0.94, *p* = .33, and the interaction, *F*(2, 189) = 1.56, *p* = .21. Removing adolescents who were not attracted to either sex did not change these findings. Controlling for pubic hair and physical developmental statuses also did not change these findings.

In terms of heterosexual expectations, the main effects of sampling group (gender nonconforming, gender conforming, or control), romantic attraction group (same-sex attracted or exclusively other-sex attracted), and their interaction were non-significant in boys, *F*(2, 183) = 0.22, *p* = .80; *F*(1, 183) = 0.47, *p* = .49; and *F*(2, 183) = 0.78, *p* = .46, respectively. In girls, the main effect of sampling groups was marginally significant, *F*(2, 179) = 2.97, *p* = .05. Planned contrasts indicated that gender nonconforming girls (*M* = 2.90, *SD* = 0.68) reported significantly fewer heterosexual expectations than control girls (*M* = 3.24, *SD* = 0.68), *p* = .02; however, there was no significant difference in heterosexual expectations between gender nonconforming and gender conforming girls (*M* = 3.15, *SD* = 0.59), *p* = .12. Removing adolescents who were not attracted to either sex did not change these findings. Controlling for pubic hair and physical developmental statuses also did not change these findings.

Among same-sex attracted youth (all non-exclusively attracted in the current study), after controlling for other-sex romantic attractions, same-sex romantic attractions were not significantly linked to other-sex sexual activities or heterosexual expectations, regardless of sex (Table 3). In contrast, after controlling for same-sex romantic attractions, other-sex romantic attractions were significantly positively associated with other-sex sexual activities and heterosexual expectations in girls, but with neither in boys (Table 3).

**Table 3:** Multivariate regressions predicting other-sex sexual activities and heterosexual expectations in same-sex attracted boys and girls

	Other-sex sexual activities		Heterosexual expectations	
	*B*	Bootstrapped 95 % CI	*B*	Bootstrapped 95 % CI
Boys				
Same-sex romantic attractions	0.69	−0.57, 3.00	0.10	−1.56, 0.95
Other-sex romantic attractions	0.22	−0.09, 0.46	0.17	−0.25, 0.82
*n*	22		19	
*R* ^2^	.40		.11	
Girls				
Same-sex romantic attractions	−0.15	−1.29, 0.87	−0.62	−1.12, 0.03
Other-sex romantic attractions	0.99	0.69, 1.33	0.31	0.02, 0.52
*n*	28		26	
*R* ^2^	.56		.20	

*Note* A bootstrapped 95 % CI that excludes 0 indicates a significant regression coefficient at .05 level, two-tailed. All same-sex attracted adolescents were non-exclusively attracted in the current sample

### Childhood Sex-Typed Behavior and Same-Sex Attractions

This analysis investigated the relationship between childhood sex-typed behavior (indicated by sampling groups) and same-sex romantic attractions. As a comparison, the differences across sampling groups of other-sex romantic attractions were also examined.

First, chi square tests were conducted to test for differences in the distributions of romantic attraction groups across sampling groups. Contrary to our hypothesis, a 2 (same-sex attracted: no or yes) × 3 (sampling group: gender nonconforming, gender conforming, or control) chi square test indicated that the percentage of same-sex attracted adolescents did not differ significantly for the three sampling groups in boys, χ^2^(2, *n* = 197) = 4.70, *p* = .10, or in girls, χ^2^(2, *n* = 195) = 2.09, *p* = .35.

Additional 2 (exclusively other-sex attracted: no or yes) × 3 (sampling group: gender nonconforming, gender conforming, or control) chi square tests indicated that the percentages of exclusively other-sex attracted youth did not differ significantly by sampling group in girls, χ^2^(2, *n* = 195) = 2.49, *p* = .29. In boys, however, there were group differences in exclusive other-sex romantic attractions, χ^2^(2, *n* = 197) = 6.72, *p* = .04. Adolescent boys who were in the gender conforming group at age 3.5 years were more likely to be exclusively attracted to the other sex (*n* = 61, 94 % of gender conforming boys) than those who were in the control group (*n* = 43, 80 % of control boys), χ^2^(1, *n* = 119) = 5.41, *p* = .02; however, the gender nonconforming group (*n* = 62, 79 % of gender nonconforming boys) had a similar percentage of exclusively other-sex attracted youth to the control group, χ^2^(1, *n* = 132) < 1.

Excluding adolescents who reported no attractions, however, eliminated the significant differences in being exclusively other-sex attracted or not across sampling groups in boys. Specifically, 2 (romantic attraction group: same-sex attracted or exclusively other-sex attracted) × 3 (sampling group: gender nonconforming, gender conforming, or control) chi square tests found no significant associations, in boys, χ^2^(2, *n* = 189) = 5.08, *p* = .08, or in girls, χ^2^(2, *n* = 181) = 2.31, *p* = .31. Because no adolescents reported exclusive same-sex attractions in the current sample, it was not possible to investigate the relationship of exclusive same-sex attractions to childhood sex-typed behavior.

Next, multivariate analyses of variance (MANOVAs) were conducted to analyze the effect of sampling group on the intensity of romantic attractions, followed by separate ANOVAs and planned contrasts on same- and other-sex attractions. Pillai’s Trace was significant in boys, *V* = 0.06, *F*(4, 388) = 2.95, *p* = .02, reflecting a significant effect of sampling groups on other-sex attractions, *F*(2, 194) = 3.32, *p* = .04, but not on same-sex attractions, *F*(2, 194) = 1.66, *p* = .19. Planned contrasts on other-sex attractions revealed that, compared with boys in the gender nonconforming group (*M* = 1.59, *SD* = 0.97), gender conforming boys reported more frequent other-sex attractions (*M* = 1.96, *SD* = 0.95), *p* = .02, but gender nonconforming boys did not report significantly different intensity of other-sex attractions than control boys (*M* = 1.66, *SD* = 1.03), *p* = .71.

After excluding boys with no attractions, however, the effect of sampling groups on other-sex attractions was not statistically significant, *F*(2, 186) = 2.57, *p* = .08. Planned contrasts indicated, however, that the significant difference between gender nonconforming (*M* = 1.66, *SD* = 0.93) and gender conforming boys (*M* = 1.99, *SD* = 0.93) remained, *p* = .03, while the former did not significantly differ from control boys (*M* = 1.73, *SD* = 0.99), *p* = .65. Controlling for pubic hair and physical developmental statuses, however, eliminated the significant findings regarding other-sex attractions, Pillai’s Trace *V* = 0.04, *F*(4, 344) = 1.56, *p* = .19.

In girls, Pillai’s Trace was not significant, *V* = 0.02, *F*(4, 384) = 1.19, *p* = .32, indicating that there was no significant differences across sampling groups for same- or other-sex romantic attractions. Excluding girls with no attractions did not alter these finding. Controlling for pubic hair and physical developmental statuses also did not alter these findings.

## Discussion

Results of this study suggest that same-sex romantic attractions among 13-year-old adolescents differ from those in adults. This conclusion is suggested by the lack of a significant sex difference in the non-exclusivity of same-sex attractions and a lack of consistently significant relations between childhood sex-typed behavior and emerging same-sex attractions.

### Non-Exclusive Patterns of Same-Sex Attractions

Several findings highlight the non-exclusive feature of emerging same-sex attractions. Strikingly, despite our over-sampling of gender nonconforming children, none of the adolescents in our sample reported exclusively same-sex romantic attractions. This finding of predominant non-exclusive attractions for adolescents with same-sex attractions resembles prior findings for adolescents’ contemporaneous reports, where non-exclusive attractions represented the major form of same-sex sexuality during early adolescence (Austin et al., 2009; Remafedi et al., 1992). Another unexpected finding in the present study is that boys were no less likely to report non-exclusive attractions than girls during early adolescence. This finding of no significant sex difference contrasts with findings from Austin et al.

The current study expanded on previous studies by analyzing non-exclusivity at an individual level. This was done by separately assessing the intensities of same- and other-sex attractions and analyzing the relations of same-sex attractions with other-sex sexual activities and heterosexual expectations. Same- and other-sex attractions were positively correlated in participants attracted to both sexes. Further, being attracted to the same sex was not associated with reduced other-sex sexual activities or heterosexual expectations and, among same-sex attracted youth, the intensity of same-sex attractions was not negatively related to the other-sex sexuality components. These findings suggest that at least some same-sex attracted young adolescents may be similar to their other-sex attracted peers in their romantic fantasies, sexual behaviors, and expectations for the future (Diamond et al., 2015; McClelland, Rubin, & Bauermeister, 2015).

Notably, all the young adolescents with non-exclusive attractions in the present study were more attracted to the other sex than to the same sex. Interpreting and reconciling weak same-sex attractions with stronger other-sex attractions may posit unique developmental challenges to these non-exclusively attracted young adolescents. Likewise, although not observed in this study, some adolescents may experience mild same-sex attractions with weak other-sex attractions. How would differences in the intensities of attractions affect a young adolescent’s sexual activities and self-perception of sexuality? And how do these processes influence psychosexual development and well-being? These are questions for future investigations.

### Early Same-Sex Attractions May Not Signal Same-Sex Orientations

This study also found that neither the prevalence nor intensity of emerging same-sex attractions related significantly and consistently to childhood gender nonconformity. This finding was unexpected, because prior evidence suggests that childhood gender nonconformity is significantly and consistently associated with increased same-sex orientations in adulthood (Bailey & Zucker, 1995; Drummond et al., 2008; Green, 1987; Rieger et al., 2008; Singh, 2012; Steensma et al., 2013; Wallien & Cohen-Kettenis, 2008; Zucker & Bradley, 1995). The inconsistent results are unlikely to be due to methodological differences between the current study and previous ones. Although most prior research was retrospective or prospective work with clinically referred samples, and the current study sampled participants from a general population and followed them prospectively, another longitudinal population-based study (Steensma et al., 2013) observed a significant relation between gender nonconformity before age 12 and same-sex attractions in adulthood. In addition, although prior studies often defined “childhood” as before 12 years old, and the assessment of the sex-typed behavior in the current study took place at age 3.5 years, Rieger et al. estimated that the significant difference in gender nonconformity between heterosexual and non-heterosexual adults was evident from ages 3–4 years.

A more likely explanation than methodological differences is that emerging same-sex attractions may differ from later same-sex orientations and may not relate to childhood sex-typed behavior. The prevalence of same-sex attractions in the current sample (13 %), especially among boys, was high compared with the prevalence reported in older populations. Among 60 studies reviewed by Savin-Williams and Vrangalova (2013), the median estimated prevalence of same-sex sexuality across adolescent and adult samples was 7.8 % for boys and 13.5 % for girls. The relatively high prevalence of boys with same-sex attractions in the current sample suggests that some pre-heterosexual boys may experience same-sex attractions in early adolescence. Similar process may occur in girls as well. In a longitudinal study on a large U.S. sample, Ott, Corliss, Wypij, Rosario, and Austin (2011) reported that approximately 5 % of pre-heterosexual girls and 4 % of pre-heterosexual boys had same-sex attractions between ages 12 and 17 years. Assuming that same-sex orientations are more reliably expressed in older people, these studies suggest that some same-sex attracted young adolescents will later identify as heterosexual.

Inconsistency between early same-sex attractions and later orientations could suggest that some early same-sex attractions arise from contexts other than sexual relationships. This might be especially likely during early adolescence, when sexual impulses are weaker than in later stages of development (note the low average intensities of attractions in Table 2). Adolescent same-sex passionate friendships may serve as a context for nurturing same-sex attractions (Peplau, 2001). Young adolescent girls and boys often form intense emotional bonds with same-sex close friends, which may involve intimate physical contact and explicit sexual interests (Diamond, 2003; Diamond, Savin-Williams, & Dubé, 1999; Way, 2011). Starting from middle adolescence, however, these person-oriented same-sex attractions may be overtaken by attractions driven by sexual desires, especially among boys, when sex drive increases, the dating and mating culture becomes more salient (Carver, Joyner, & Udry, 2003), and they are pushed to distance themselves from same-sex best friends (Way, 2011). However, these suggestions regarding developmental processes are largely speculative; more research is needed to document the development of intimate relationships and sexuality across adolescence (Diamond et al., 2015).

### Limitations

A potential methodological limitation of the current study is self-report bias. It is possible that some adolescents who reported same-sex attractions were pretending (Savin-Williams & Joyner, 2014; but see Li, Katz-Wise, & Calzo, 2014). Had this been true, however, it would have been likely to produce some reports of exclusive same-sex romantic attractions. It is also possible that adolescents who experienced exclusive same-sex romantic attractions might have intentionally hidden them, for reasons of stigma associated with same-sex sexuality. While it is not possible to completely rule out this possibility, the use of computerized self-interviews should have enhanced participants’ disclosure of sensitive private information (Turner et al., 1998), as should the reduced salience of the sensitive questions, which were embedded in a larger study protocol. Future research might use automatic, nonintrusive measures of romantic and sexual attractions to further reduce self-report bias.

The sample size might also affect the results of the present study. For instance, a larger sample might have produced some exclusively same-sex attracted young adolescents. Although we attempted to address this possibility by oversampling gender nonconforming children, future studies might usefully replicate the findings reported here in larger samples of young adolescents.

## Conclusion

The current study suggests that, although same-sex romantic attractions emerge early, they are typically non-exclusive in early adolescence and they differ from later same-sex orientations. The present study underscores the importance of studying the development of adolescent sexuality contemporaneously. Following adolescents from early adolescence could help reveal the developmental trajectories of not only non-heterosexual orientations but also heterosexual orientation, especially when both processes involve early same-sex attractions. These early attractions may originate from sexual desires, but also possibly from emotional ties.

### Electronic supplementary material

Below is the link to the electronic supplementary material.

Supplementary material 1 (DOC 109 kb)

Supplementary material 2 (DOC 127 kb)

Supplementary material 3 (DOC 43 kb)

## Appendix

### Measurement Validity of Romantic Attractions (Adapted from Storms, 1980)

A series of single-group confirmatory factor analyses (CFAs) were conducted, using R package “lavaan” (Version 0.5–17; Rosseel, 2012), to examine the latent correlation of romantic attractions at 13 years of age (Table A1, Models 1–3). Results indicated that, in the whole sample, same-sex romantic attractions were neither orthogonal or opposite to other-sex romantic attractions; rather, the two types of romantic attractions were positively correlated, *r*(390) = .21, bootstrapped 95 % CI = [.11, .30]. Furthermore, multi-group CFAs revealed that the positive correlation between same- and other-sex romantic attractions was similar in both sex groups (Table A1, Models 4–10; Fig. A1), suggesting that boys and girls did not significantly differ in the non-exclusivity of romantic attractions.

### Scoring Adolescent Sexual Activities Index by Item Response Theory

Items in the Adolescent Sexual Activities Index (Hansen et al., 1999) were fitted using the Rasch model, a one-parameter logistic (1PL) model derived from the item response theory (IRT) (Embretson & Reise, 2000). IRT assumes that each item varies in difficulty; the more difficult an item is the less likely an individual is going to score on that item. Likewise, for an item at a certain difficulty level, an individual whose ability is above that item difficulty is more likely to score on this item than an individual with a lower ability. Applying to the context of adolescent sexual activities, starting from hugging, each activity becomes less popular in the young adolescent population, and only individuals who have an exceptionally high sexual activity level will reach the last stage—sexual intercourse (Fig. A2, upper panel). Therefore, “hugging” had the lowest item difficulty (most popular), while “sexual intercourse” had the highest item difficulty (least popular). The probability of an individual at a given sexual activity level engaging in various sexual activities decreases with decreased item difficulty (popularity) from hugging to sexual intercourse (Fig. A2, upper panel).

The Scale Information Function reflects the total psychometric information the items provide. It can be regarded as a measure of scale reliability. IRT measurement becomes more accurate (i.e., yields smaller confidence intervals) when the scale information increases. Regarding the ASAI, scale information peaked three times, corresponding to three stopping points at “spending time alone,” “touching under clothes,” and “touching or foundling a boy’s/girl’s private parts” (Fig. A2, lower panel). Therefore, IRT scoring is the most accurate for participants who scored at these sexual activity levels. The R package “ltm” (Version 1.0-0; Rizopoulos, 2006) was used to perform the IRT model fitting and scoring, using Rasch model with Gauss-Hermite quadrature rule and BFGS algorithm to fit the data and expected a posteriori method to calculate individual scores. Because a 2PL model, with the additional item discrimination parameter than the 1PL Rasch model, did not significantly improve the model fit of the latter, χ^2^(11) = −29.28, *p* > .99, the 1PL Rasch model was retained and used to score individual adolescent sexual activity level.

## Footnotes

1 These classifications differ from some prior reports on this sample (e.g., Golombok, Rust, Zervoulis, Golding, & Hines, 2012), where children have been grouped as masculine, control, and feminine, because hypotheses for the current study required combining masculine boys and feminine girls into a gender conforming group and feminine boys and masculine girls into a gender nonconforming group.
