# Supplementary material for: In Search of Emerging Same-Sex Sexuality: Romantic Attractions at Age 13 Years
Source: Arch Sex Behav. 2016 Apr 18;45:1839–49. doi: 10.1007/s10508-016-0726-2 (PMC4987389; doi:10.1007/s10508-016-0726-2)
Supplement: Supplementary file 1 — Supplementary material 1 (DOC 109 kb) [file 10508_2016_726_MOESM1_ESM.doc]

*Figure A1.* Final fitted model (Model 10 in Table A1) using marker identification with unstandardized estimates, adjusted for sampling groups by childhood sex-typed behavior (not shown). The correlated residual of the second and third indicators was freely estimated according to modification indices. Residual variances and indicator intercepts are not shown. Dashed lines demonstrate non-significant paths, *p*s < .05. Asterisk marks denote fixed estimates. From bottom to top: The boxes (indicators) labeled from 1 to 14 correspond to the items in the Erotic and Response and Orientation Scale (see the table under the graph) (Storms, 1980). The numbers on the directed path from the circles (latent [indirectly measured] constructs) denote factor loadings. The first item (Items 1 and 8) of each latent construct was used as corresponding reference. Estimates on the directed paths from the triangle denote latent means of latent constructs. Double arrows indicate latent variances/ covariance. Unless otherwise indicated, all the estimates marked in the graph did not significant differ between the sexes. Notably, the latent covariance between same-sex and other-sex romantic attractions was significantly larger than 0 and did not significantly differ between girls and boys. ♀ = girls; ♂ = boys.

*
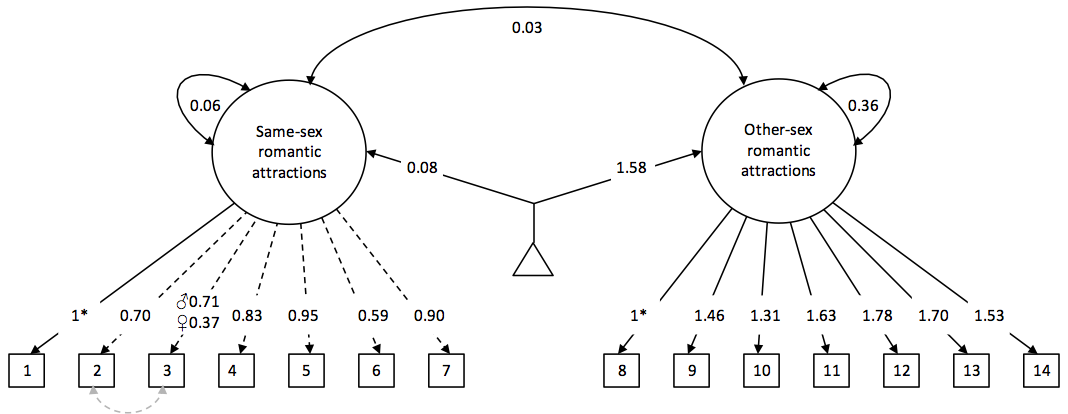
*

| 1 | How often have you noticed that a same-sex person you have seen or met for the first time is physically attractive to you? | 8 | How often have you noticed that an other-sex person you have seen or met for the first time is physically attractive to you? |
| --- | --- | --- | --- |
| 2 | How often have you had romantic feelings whilst looking at a same-sex person? | 9 | How often have you had romantic feelings whilst looking at an other-sex person? |
| 3 | How often have you felt excitement from touching or being touched by a same-sex person? | 10 | How often have you felt excitement from touching or being touched by an other-sex person? |
| 4 | How often have you thought about what it would be like to have a romantic experience with a same-sex person? | 11 | How often have you thought about what it would be like to have a romantic experience with an other-sex person? |
| 5 | How often have you felt a desire to have romantic experience with a particular same-sex person you know? | 12 | How often have you felt a desire to have romantic experience with a particular other-sex person you know? |
| 6 | How often have you daydreamed about having a romantic experience with a same-sex person? | 13 | How often have you daydreamed about having a romantic experience with an other-sex person? |
| 7 | How often have you dreamed at night about having a romantic experience with a same-sex person? | 14 | How often have you dreamed at night about having a romantic experience with an other-sex person? |
