# Supplementary material for: In Search of Emerging Same-Sex Sexuality: Romantic Attractions at Age 13 Years
Source: Arch Sex Behav. 2016 Apr 18;45:1839–49. doi: 10.1007/s10508-016-0726-2 (PMC4987389; doi:10.1007/s10508-016-0726-2)

*Figure A2.* Fitting sexual activity items into a 1PL Rasch model: (upper) Item Response Curves; (lower) Scale Information Function.


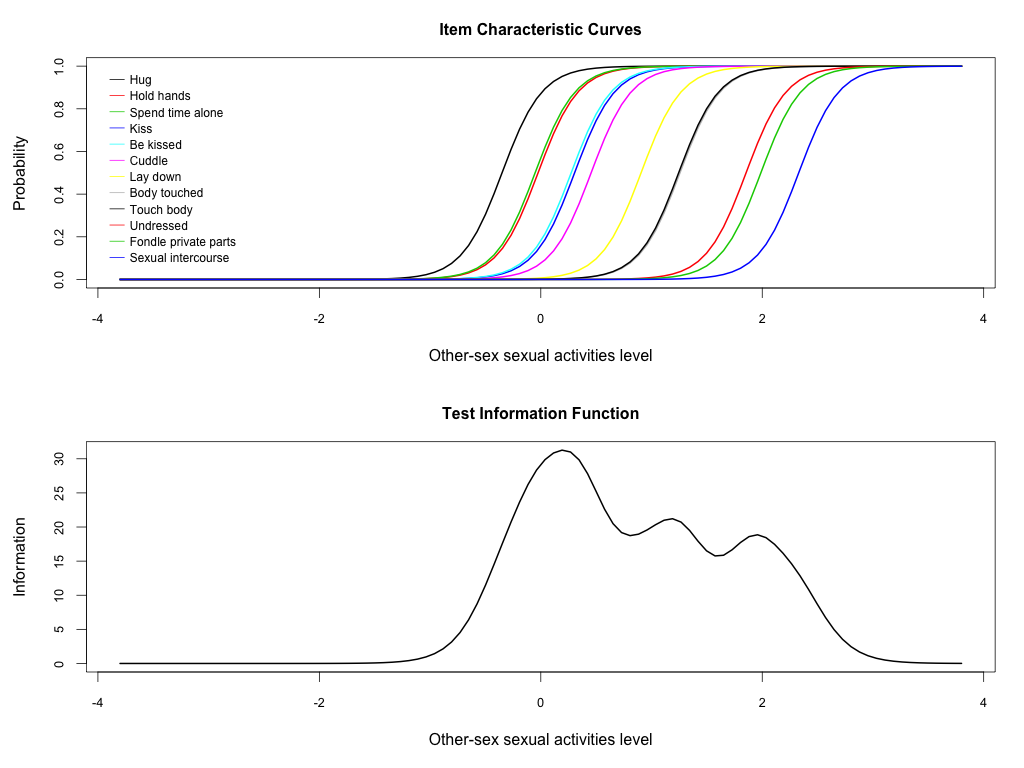

Supplement: Supplementary file 2 — Supplementary material 2 (DOC 127 kb) [file 10508_2016_726_MOESM2_ESM.doc]
