# Supplementary material for: In Search of Emerging Same-Sex Sexuality: Romantic Attractions at Age 13 Years
Source: Arch Sex Behav. 2016 Apr 18;45:1839–49. doi: 10.1007/s10508-016-0726-2 (PMC4987389; doi:10.1007/s10508-016-0726-2)
Supplement: Supplementary file 3 — Supplementary material 3 (DOC 43 kb) [file 10508_2016_726_MOESM3_ESM.doc]

Table A1

*Model Fit Statistics for the Latent Correlation and Sex Comparisons of Romantic Attractions (n* = 390)

| Model tested | χ^2^ | *df* | Δχ^2^ | Δ*df* | *p* | RMSEA | RMSEA 90% CI | CFI | ΔCFI | Pass? |
| --- | --- | --- | --- | --- | --- | --- | --- | --- | --- | --- |
| Tests for latent correlation of romantic attractions | | | | | | | | | | |
| 1. Baseline model | 207.39 | 99 | — | — | — | — | —, — | — | — | — |
| 2. One-dimensional model | 758.37 | 100 | 550.98^a^ | 1^a^ | <.001^a^ | — | —, — | — | — | No |
| 3. Orthogonal model | 220.58 | 100 | 13.19^a^ | 1^a^ | <.001^a^ | — | —, — | — | — | No |
| Invariance tests for sex comparisons | | | | | | | | | | |
| 4. Configural invariance | 355.63 | 198 | — | — | — | .060 | .049, .071 | .969 | — | Yes |
| 5. Weak invariance | 414.31 | 210 | — | — | — | .071 | .061, .081 | .955 | .014^b^ | No |
| 6. Weak invariance–with free estimates | 370.86 | 209 | — | — | — | .063 | .052, .073 | .964 | .005^b^ | Yes |
| 7. Strong invariance | 410.73 | 221 | — | — | — | .066 | .056, .076 | .958 | .006^c^ | Yes |
| Sex comparisons of latent variances/covariances and means | | | | | | | | | | |
| 8. Homogeneity of latent variances and covariances | 421.34 | 224 | 10.61^d^ | 3^d^ | .014^d^ | — | —, — | — | — | Yes |
| 9. Means–omnibus | 423.81 | 226 | 2.47^e^ | 2^e^ | .291^e^ | — | —, — | — | — | Yes |
| 10. Final model | 423.81 | 226 | — | — | — | .067 | .057, .077 | .956 | — | Yes |

*Note.* For the tests of latent correlation of romantic attractions and sex comparisons of latent variances/covariances and means, given the power of the sample size, a *p*-value less than .005 was used (Little, 2013). For the measurement model tests of invariance, a change in CFI of .01 or less was used as the passing criterion (Little, 2013).

Model 1: One-group model; the covariance between same-sex romantic attractions and other-sex romantic attractions was freely estimated.

Model 2: One-group model; the covariance between same-sex romantic attractions and other-sex romantic attractions was restricted to -1.

Model 3: One-group model; the covariance between same-sex romantic attractions and other-sex romantic attractions was restricted to 0.

Model 4: Two-group model, separated by sex; the covariance between same-sex romantic attractions and other-sex romantic attractions was freely estimated.

Model 5: Further constrained Model 4 by setting corresponding factor loadings equal between boys and girls.

Model 6: All other corresponding factor loadings were set equal between boys and girls, except for the item “felt excitement from touching or being touched by a same-gender person” on the same-gender romantic attractions dimension. The factor loading of this item was larger in boys than that in girls (Fig. A1).

Model 7: Further constrained Model 6 by equating corresponding indicator intercepts.

Model 8: Further constrained Model 7 by equating corresponding latent variances and covariances.

Model 9: Further constrained Model 8 by equating corresponding latent means.

Model 10: Same as Model 9 (Fig. A1).

^a^ Compared to Model 1.

^b^ Compared to Model 4.

^c^ Compared to Model 6.

^d^ Compared to Model 7.

^e^ Compared to Model 8.
